# Mesenchymal stem cell-derived small extracellular vesicles-loaded GelMA microspheres enhance diabetic wound healing by promoting M2 macrophage polarization through p38 MAPK inhibition

**DOI:** 10.1016/j.mtbio.2025.102423

**Published:** 2025-10-17

**Authors:** Weizhao Li, Jiajia Chen, Lu Yu, Lu Ding, Xiaoying Zhang, Leping Yan, Ming Shi

**Affiliations:** a Department of Scientific Research Center, The Seventh Affiliated Hospital, Sun Yat-Sen University, Shenzhen, 518107, China; b Department of Infectious Diseases and Public Health, Jockey Club College of Veterinary Medicine and Life Sciences, City University of Hong Kong, Kowloon, 999077, Hong Kong, China; c Department of Clinical Laboratory, The Seventh Affiliated Hospital, Sun Yat-Sen University, Shenzhen, 518107, China; d Department of Health Management Center, The Seventh Affiliated Hospital, Sun Yat-Sen University, Shenzhen, 518107, China; e Guangdong Provincial Key Laboratory of Digestive Cancer Research, The Seventh Affiliated Hospital, Sun Yat-sen University, Shenzhen, 518107, China

**Keywords:** Small extracellular vesicles, Macrophage polarization, p38 MAPK pathway, Diabetic wound healing

## Abstract

Diabetic foot ulcers (DFUs) represent a serious complication of diabetes, typically exhibiting persistent inflammation and impaired tissue repair. Although small extracellular vesicles derived from mesenchymal stem cells (MSC-sEVs) possess therapeutic potential for diabetic wound repair by modulating inflammatory responses, their regulatory mechanisms and limited *in vivo* retention remain challenges. Here, we developed MSC-sEVs-loaded gelatin methacryloyl (GelMA) microspheres (sEVs@MS) as a therapeutic dressing for diabetic wounds. MSC-sEVs were characterized and found to induce M1-to-M2 polarization in lipopolysaccharide (LPS)-treated bone marrow-derived macrophages (BMDMs), significantly downregulating iNOS and TNF-α while upregulating CD206 and IL-10 *in vitro*. RNA sequencing analysis identified *Mapk14* and *Nfkbia* as key regulators within the p38 MAPK signaling pathway, with their expression levels significantly decreased following MSC-sEVs treatment. Consistent with these findings, western blot confirmed that MSC-sEVs effectively inhibit the p38 MAPK pathway with decreased phosphorylation of p38 and MAPKAPK2. To achieve sustained delivery of MSC-sEVs, we fabricated lyophilized GelMA microspheres and loaded the vesicles using a rehydration-induced swelling strategy. *In vivo* experiments demonstrated that both sEVs and sEVs@MS treatments enhanced M2 macrophage polarization and reduced inflammation, whereas sEVs@MS prolonged MSC-sEVs retention at the wound site for 7 days, thereby promoting wound closure by day 28. Histological analysis further confirmed that sEVs@MS improved epidermal regeneration and collagen deposition, ultimately accelerating wound repair in diabetic rats. Collectively, these findings establish sEVs@MS as an effective therapeutic strategy for diabetic wounds treatment.

## Graphical abstract

This study introduces a therapeutic strategy for diabetic wound healing by delivering mesenchymal stem cell–derived small extracellular vesicles (MSC-sEVs) via GelMA microspheres. MSC-sEVs, isolated from bone marrow mesenchymal stem cells, were internalized by M1 macrophages and promoted their polarization toward an M2 phenotype through inhibition of the p38 MAPK pathway. Encapsulation within lyophilized GelMA microspheres (sEVs@MS) enabled sustained release of MSC-sEVs. Applied to diabetic wounds, this controlled release suppressed pro-inflammatory cytokines, enhanced M2 macrophage accumulation, and facilitated tissue repair. By combining the immunomodulatory properties of MSC-sEVs with the structural and functional advantages of GelMA microsphere dressings, this approach accelerated diabetic wound healing.

**Figure:**
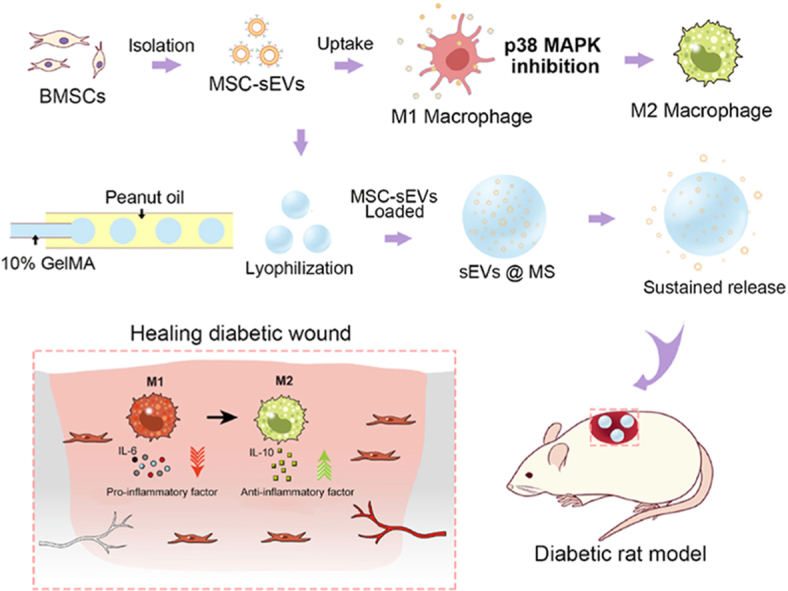

## 1 Introduction

Approximately 18.6 million diabetic patients experience diabetic foot ulcers (DFUs) globally, and approximately 20 % requiring lower extremity amputations, which severely impacts patients’ quality of life [1,2]. Investigating the pathogenesis of DFUs and developing effective therapeutic strategies for diabetic wound are crucial.

Current clinical management of DFUs involves multimodal strategies: strict glycemic regulation, necrotic tissue removal, and the application of moisture-retentive dressings [3]. Other approaches include vascular interventions or pharmacotherapy to enhance circulation, with surgical options such as amputation or skin grafting reserved for severe cases [4]. However, persistent inflammatory responses in diabetic wounds lead to deep tissue injury and chronic ulceration [5]. Conventional dressings exhibit limited efficacy in modulating subdermal inflammatory microenvironments, highlighting the urgent need for advanced dressings with targeted immunomodulatory properties [6,7].

Wound healing progresses through four phases: hemostasis, inflammation, proliferation, and remodeling [8]. Macrophages play pivotal roles throughout these stages, and their depletion impairs granulation tissue formation, angiogenesis, and wound closure. [9]. During normal healing, approximately 85 % of macrophages exhibit the M1 phenotype in early stages, transitioning to 80–85 % M2 polarization between days 5–7 [10]. However, diabetic wounds demonstrate impaired M1-to-M2 macrophage transition, leading to non-healing wound [11,12]. These investigations highlight macrophages as essential targets for diabetic wound recovery.

MSC-sEVs are promising acellular therapies with potent immunoregulatory and tissue-repairing properties [13,14]. Increasing evidence indicates that MSC-sEVs can promote macrophage shift to M2 phenotype, thereby promoting diabetic wound recovery [15]. However, rapid clearance and insufficient local retention at the wound site after direct administration significantly limit their therapeutic efficacy [16,17]. To address these challenges, researchers have incorporated MSC-sEVs into hydrogel systems, which provide a protective matrix for sustained release and enhanced therapeutic effects in diabetic wounds [18,19]. Among these hydrogels, gelatin methacryloyl (GelMA) demonstrates superior biocompatibility, making it an optimal biomaterial for the sustained delivery of sEVs in diabetic wound therapy [18,20,21]. For instance, biotin-modified GelMA hydrogels loaded with MSC-sEVs have been shown to promote M2 macrophage polarization and contribute to improved tissue repair in diabetic wounds [22]. Despite these advances, the mechanisms underlying MSC-sEVs–induced macrophage polarization remain to be fully elucidated. In addition, conventional bulk hydrogels may hinder uniform sEVs release and limit deep tissue penetration, thereby reducing their efficacy in irregular wound sites. Our previous studies have shown that microsphere-based dressings can locally deliver cytokines and stem cells with improved retention and spatial adaptability [23,24]. Based on this strategy, GelMA microsphere dressings were designed to enable sustained local release of MSC-sEVs, and their therapeutic mechanisms in diabetic wound healing were systematically investigated.

Herein, we employed microfluidic technology to fabricate the GelMA microspheres. Following the isolation and characterization of MSC-sEVs, cell-based assays were carried out to investigate their effects on LPS-induced M1 macrophage polarization. Subsequent RNA-seq and multiplex cytokine profiling identified significant gene expression and secretion alterations, with pathway enrichment analysis informing further mechanistic validation. sEVs@MS was then prepared by thoroughly encapsulating MSC-sEVs into the lyophilized GelMA microspheres. The release profile and degradation rate were evaluated *in vitro*. In addition, diabetic wounds were modeled to further examine the healing potential of MSC-sEVs loaded microspheres. This study not only constructed an innovative MSC-sEVs-based microsphere dressing but also elucidated its mechanisms in inflammatory regulation and diabetic wound repair.

## 2 Materials and methods

### 2.1 Cell culture

Rat bone marrow mesenchymal stem cells (BMSCs) were purchased from Shanghai GuanDao Biological Engineering Co., Ltd, cultured in complete Dulbecco's modified Eagle medium (DMEM)/F12 medium (Gibco, USA) supplied with 10 % fetal bovine serum (FBS, 04–001-1ACS, BI, Israel) and 1 % penicillin/streptomycin (P/S). The BMSCs were maintained in a 37 °C incubator with 5 % CO_2_.

### 2.2 BMDMs isolation and identification

BMDMs from Sprague-Dawley (SD) rats were isolated as previous [25]. Briefly, bone marrow cells from leg bones, were erythrocytes lysed, filtered with 70 μm filter, and maintained in complete medium with 30 ng/mL M-CSF (HY-p7247, MCE, USA) for 7 days. The culture medium was refreshed every 3 days, and the adherent cells were BMDMs.

To identify BMDMs, CD11b and CD68 were measured by flow cytometry. Cells were incubated with CoraLite® Plus 647 anti-rat CD11b (CL647-65606, Proteintech, China) and PE anti-rat CD68 antibody (201003, Biolegend, USA). Propidium Iodide (537060, Sigma, Germany) was applied to stain the dead cells. Collect data with a CytoFLEX flow cytometer (Beckman, Germany) and analyzed with FlowJo (v10.9).

### 2.3 Isolation of MSC-sEVs from BMSCs

MSC-sEVs were isolated using the ultracentrifugation method [26]. BMSCs at ∼80 % density was cultured in pure DMEM/F12 for 48 h after phosphate-buffered saline (PBS) rinsing. Supernatant was centrifuged, filtered, and ultracentrifuged to isolate purified MSC-sEVs in Dulbecco's PBS (DPBS).

### 2.4 Characterization of MSC-sEVs

#### 2.4.1 Transmission electron microscopy (TEM)

Two μL of MSC-sEVs suspension was placed on a carbon-filmed copper grid for 3–5 min, stained with 2 % phosphotungstic acid and air-dried. The samples were characterized with TEM (HT7800, HITACHI, Japan).

***2.4.2*** ***Nanoparticle tracking analysis (NTA)***

MSC-sEVs size were measured using NTA with ZetaView (Particle Metrix, Germany). Prior to measurement, MSC-sEVs were diluted in PBS based on preliminary titrations to ensure accurate tracking. The particle size distribution data was generated by NTA software, which analyzed the Brownian motion of particles to calculate the particle size based on the Stokes-Einstein equation.

### 2.5 BMDMs uptake Dil-labeled MSC-sEVs

For internalization assay, MSC-sEVs, labeled with 20 μg/mL Dil dye (D3911, ThermoFisher, USA) solution at 37 °C for 20 min, were centrifuged at 10,000×*g* for 5 min, and co-cultured with BMDMs for 1 day. BMDMs were stained with phalloidin (A12379, Invitrogen, 1:200) and DAPI (1:5000; Sigma). Images were obtained by confocal microscopy (CLSM, Zeiss, Germany). The overlap of red fluorescence of Dil-MSC-sEVs and green fluorescence of actin cytoskeleton represents that MSC-sEVs were uptaked by BMDMs.

### 2.6 *In vitro* BMDMs treatment

BMDMs were cultured in 10 cm dishes for 7 days. The experiment was divided into three groups: the blank (culture for 72 h without LPS and MSC-sEVs treatment), LPS (100 ng/mL, 24 h and then PBS treated for 48 h), LPS + MSC-sEVs (100 ng/mL LPS treated 24 h and then 30 μg/mL MSC-sEVs treated 48 h). The supernatants of each group were collected for cytokine detection. BMDMs were rinsed twice with PBS to eliminate culture medium and lysed with TRIzol or radio immunoprecipitation assay (RIPA) for total RNA or protein extraction. For immunofluorescent staining, BMDMs were incubated in a confocal petri dish under the same condition as above. Rinse BMDMs with PBS and fixed with 4 % paraformaldehyde (PFA).

### 2.7 RNA isolation and RT-qPCR analysis

Total RNA was isolated using an RNA extraction kit (RN001, ESScience, China), and complementary DNA (cDNA) was synthesized with a reverse transcription kit (RT001, ESScience, China). Quantitative real-time PCR (RT-qPCR) was performed on a CFX96 system (Bio-Rad, USA) to determine the relative mRNA levels of *Tnf* (TNF-α), *Il10* (IL-10), *Nos2* (iNOS), and *Mrc1* (CD206), with primers listed in Table 1. Gene expression was normalized to β-Actin and calculated using the 2^−ΔΔCt^ method.

**Table 1:** Primer sequences.

Gene		Sequence (5′ to 3′)
β-Actin	Forward	GCTGTGCTATGTTGCCCTAGACTTC
	Reverse	GGAACCGCTCATTGCCGATAGTG
iNOS	Forward	GAGACGCACAGGCAGAGGTTG
	Reverse	AGCAGGCACACGCAATGATGG
CD206	Forward	GGCACCAGGCAAGAGCAAGC
	Reverse	TCCAATCCAGAGTCCAGAGGTCAAG
TNF-α	Forward	AAAGGACACCATGAGCACGGAAAG
	Reverse	CGCCACGAGCAGGAATGAGAAG
IL-10	Forward	ACTGCTATGTTGCCTGCTCTTACTG
	Reverse	TGGGTCTGGCTGACTGGGAAG

### 2.8 Magnetic luminex screening assay

Cytokine levels in supernatants (three groups: blank, LPS, and MSC-sEVs, n = 3) were detected with the Rat Cytokine kit (12005641, BIO-RAD, USA) and analyzed on a Luminex 200 system (Thermo Fisher, USA).

### 2.9 RNA-seq and analyses

RNA-seq analysis was performed for three experimental groups (blank, LPS, and MSC-sEVs) by AccuraMed Company (Shanghai, China). Briefly, RNA libraries were prepared using the TruSeq Stranded mRNA Kit (Illumina, USA), quantified with Qubit, and assessed for quality using a Bioanalyzer. Libraries were sequenced on an Illumina NovaSeq 6000 platform (150 bp paired-end), followed by adapter trimming with Trim Galore and alignment to the rat reference genome (RGSC6.0) using HISAT2. Gene-level counts were obtained with featureCounts (v2.0.3), and differential expression analysis was conducted using DESeq2 (v1.30.0). Data visualization was performed in R (v4.0.3), and functional enrichment analysis of differentially expressed genes was carried out using ClusterProfiler (v4.2.2).

### 2.10 Fabrication and characterization of GelMA microspheres

To achieve sustained local release of MSC-sEVs, GelMA microspheres were fabricated using microfluidic technology [27]. The internal phase, composed of a 10 % (w/v) GelMA solution in PBS containing 0.25 % LAP, was maintained at 37 °C and delivered into the microfluidic device at a flow rate of 5 μL/min. Simultaneously, peanut oil was injected at 50 μL/min as the continuous phase. The droplets were collected and crosslinked under blue light (405 nm) for 1 min, and centrifuged. The microspheres were washed thrice with 5 % FBS, freeze-dried, and stored at −80 °C.

### 2.11 Swelling ratio measurement

To evaluate the swelling behavior of lyophilized GelMA microspheres, 10 mg freeze-dried microspheres were placed into 0.2 ml microcentrifuge tubes with gradient volumes of PBS (20, 40, 80, and 100 μL) and incubated at 37 °C for 1 h. Photographs were taken to observe the extent of microsphere swelling and hydration. Upon determining the optimal volume that allows microspheres to reach swelling equilibrium, tubes were centrifuged at 1000 rpm for 5 min. The PBS supernatant was removed, and any residual PBS within the tubes was gently absorbed with filter paper. The wet weight of the rehydrated microspheres was then measured. Each group was repeated thrice. The swelling ratio was calculated by formula (1):

(1) $$\text{Swelling}\;r\text{atio}\;\left(\%\right)=\left(W_{s}-W_{d}\right)∕W_{d}\times 100$$

Where $W_{s}$ represents the weight of the microspheres after rehydration, and $W_{d}$ represents the initial dry weight of the microspheres.

### 2.12 Atomic Force Microscopy test

The mechanical stiffness of the rehydrated GelMA microspheres was assessed by Atomic Force Microscopy (AFM; NanoWizard 4XP, Bruker, USA) under liquid conditions. Measurements were performed in contact mode using a tipless silicon nitride cantilever (MLCT-O10, Type A, Bruker, Camarillo, CA) at a constant approach/retract speed of 2 μm/s and a force trigger threshold of 5 nN. The resulting force–distance curves were baseline-corrected and analyzed using JPK SPM software (Version 6.1.14, JPK Instruments AG, Germany). The Young's modulus was determined based on the Hertz contact model for a spherical elastic body, assuming a Poisson's ratio of 0.5.

To ensure measurement accuracy and reproducibility, nine testing points were selected on each microsphere surface, arranged in a 3 × 3 grid with an inter-point spacing of at least 5 μm. At each point, three repeated measurements were performed to minimize random variability. The final Young's modulus was calculated as the average of values obtained from five randomly selected microspheres.

### 2.13 Degradation test

Lyophilized microsphere samples (50 mg, n = 4 per group) were incubated with 2 mL PBS containing 1 % P/S to maintain sterility and prevent undesired GelMA degradation, and refreshed medium every 2 days. At predetermined time points, samples from each group were collected, washed thrice with deionized water, lyophilized, and weighted to determine degradation rates via formula (2):

(2) $$\text{Degradation}\;\text{rate}\;\left(\%\right)=\left(M_{0}-M_{t}\right)∕M_{0}\times 100$$

where $M_{0}$ and $M_{t}$ are the dry masses at day 0 and day t, respectively.

### 2.14 Encapsulation efficiency of MSC-sEVs

The encapsulation efficiency of MSC-sEVs within GelMA microspheres was evaluated using NTA (NanoSight NS300, Malvern, UK). Briefly, 10 mg of lyophilized microspheres were incubated with 100 μL of MSC-sEVs suspension (1 mg/mL in PBS) at 37 °C for 1 h to allow complete hydration and adsorption (sEVs@MS group). Control microspheres were incubated with PBS under identical conditions (MS group). After incubation, the supernatant was collected and its volume recorded. Particle concentrations were measured by NTA after diluting the samples to appropriate concentrations. The total number of sEVs initially added ($N_{total}$) and the number of free sEVs remaining in the supernatant ($N_{free}$) were used to calculate encapsulation efficiency using the following formula (3):

(3) $$\text{Encapsulation}\;\text{efficiency}\;\left(\%\right)=\left(N_{total}-N_{free}\right)∕N_{total}\times 100$$

Here, $N_{total}$ was determined from the initial sEVs concentration corrected for PBS background, and $N_{free}$ was corrected for background particles released from the MS group.

### 2.15 MSC-sEVs release from GelMA microspheres

sEVs@MS samples were prepared by incubating 10 mg lyophilized microspheres with 100 μL of MSC-sEVs suspension (1 mg/mL) at 37 °C for 1 h to allow full hydration and absorption. MS samples were incubated with 100 μL PBS under identical conditions as control. After incubation, the microspheres were collected for the release assay. For *in vitro* release, microspheres from both the sEVs@MS and MS groups (n = 3 per group) were suspended in 1 mL of PBS and maintained at 37 °C. At predetermined points, 0.2 mL supernatant was collected for measurement and supplied 0.2 mL fresh PBS. Particle in the supernatant were measured. The cumulative release of MSC-sEVs was calculated using formula (4):

(4) $$\text{Cumulative}\;\text{release}\;\left(\%\right)=\left[\sum_{i=1}^{n-1}C_{i}\times V+C_{n}\times V_{0}\right]/N_{loaded\;}\times 100$$

Where V is the volume of supernatant collected (0.2 mL), $V_{0}$ is the total PBS volume (1 mL), $C_{i}$ and $C_{n}$ represent the concentrations of sEVs at each time point (i = 1 to n), and $N_{loaded}$ indicates the total sEVs initially loaded in the microspheres, which was calculated based on $N_{total}$ and $N_{free}$ following the method described in Section 2.14.

### 2.16 Evaluation of sEVs@MS on macrophage internalization and polarization

To evaluate whether MSC-sEVs released from sEVs@MS can be internalized by BMDMs, Dil-labeled MSC-sEVs were first prepared and encapsulated into lyophilized GelMA microspheres. BMDMs were then co-cultured with the Dil-MSC-sEVs-loaded microspheres for 24 h and stained with phalloidin, as described in Section 2.5. The distribution of Dil-labeled sEVs within the microspheres and their internalization by BMDMs were visualized using confocal microscopy.

To assess the biological effect on macrophage, BMDMs and sEVs@MS were co-cultured in a Transwell system (3450, Corning, USA). Briefly, BMDMs were plated in the lower chamber and stimulated with 100 ng/mL LPS for 24 h. Subsequently, sEVs@MS were applied in the upper chamber for an additional 48 h of co-culture. Immunofluorescence staining for iNOS (sc-7271, 1:200, Santa Cruz, USA), CD206 (18704-1-AP, 1:200, Proteintech, China), and DAPI (1:5000, Sigma) was performed as described in later sections.

### 2.17 Animal experiments

All animal experiments were performed in accordance with protocols approved by the Institutional Animal Care and Use Committee of Top Biotechnology Company. (TOPGM-IACUC-2024-0081).

#### 2.17.1 Type I diabetes mellitus (TIDM) rat model

TIDM was induced in 8-week-old SD rats, followed by the creation of a full-thickness skin excision model according to our prior study [23]. Briefly, 1 % streptozotocin (STZ) solution (prepared in sodium citrate-hydrochloric acid buffer, 50 mg/kg) was administered through intraperitoneal injection after fast for 24 h. TIDM rats maintained on a high-fat, high-sugar diet and exhibiting blood glucose levels above 16.7 mmol/L for 3 days were chosen for the procedure. Rats were anesthetized with 3 % sodium pentobarbital, and two full-thickness dorsal skin wounds, each 1 cm in diameter, were created.

#### 2.17.2 Diabetic wounds treatment and analysis

sEVs@MS samples were prepared as follows: 10 mg of lyophilized GelMA microspheres were incubated with 100 μL of MSC-sEVs suspension (1 mg/mL) at 37 °C for 1 h to allow complete hydration and encapsulation. As a control, blank microspheres (MS) were incubated with 100 μL of PBS under the same conditions. Each sample was individually applied to the wound site.

TIDM rats were randomly assigned to four groups. The blank group received 100 μL PBS, and the sEVs group received 100 μg MSC-sEVs in 100 μL PBS through subcutaneous injection around the wound. For the MS group and sEVs@MS group, microspheres were gently applied directly onto the wound surface. Excess fluid around the wound edge was carefully removed using a sterile cotton swab. A medical sticker (Cofoe, China) was placed centered over the wound, with a rubber splint (inner diameter ∼15 mm) attached on the adhesive side of the sticker and further sealed with a transparent, waterproof film. This setup created a closed chamber over the wound area, allowing localized retention of the microspheres. To ensure stability, a self-adhesive elastic bandage was wrapped around the dorsal region to secure the dressing in place (Fig. S1).

Wound were photographed on days 3, 7, 14, and 28, and their areas were quantified using ImageJ. Wound contraction was calculated according to formula. (5):

(5) $$\text{Wound}\;\text{contraction}\;\left(\%\right)=\left(A_{0}-A_{t}\right)∕A_{0}\times 100$$

where $A_{0}$ represents the initial wound area on day 0 and $A_{t}$ indicates the wound area at the given time point.

Rats were euthanized with an overdose of 3 % sodium pentobarbital solution at determined timepoints. Skin samples (1.5 × 1.5 cm) surrounding the wound were collected and processed for paraffin embedding, followed by Hematoxylin and Eosin (H&E) and Masson's trichrome staining to evaluate tissue regeneration. Collagen deposition area was quantified by ImageJ [28].

#### 2.17.3 *In vivo* biodistribution of MSC-sEVs

MSC-sEVs were labeled with DiR (D12731, ThermoFisher, USA) as the same procedure as Dil. A total of 100 μg DiR-labeled MSC-sEVs suspended in 100 μL PBS (n = 3) were injected subcutaneously around the wound margins. Similarly, DiR-labeled sEVs@MS were applied to the wound bed. Fluorescence intensity was assessed with an imaging system (IVIS Lumina Series III), and skin fluorescence signals were captured and quantified via Living Image software (PerkinElmer, USA).

### 2.18 Immunofluorescence (IF) staining

#### 2.18.1 IF staining of cells

Cells were fixed in 4 % PFA, and permeabilized with 0.1 % Triton X-100, blocked with 3 % bovine serum albumin (BSA), and incubated overnight at 4 °C with primary antibodies, including CD206 (18704-1-AP, 1:200, Proteintech, China) and iNOS (sc-7271, 1:200, Santa Cruz, USA). After rinsed with PBS, Alexa Fluor 568/647-conjugated secondary antibodies and DAPI were applied, and images were acquired using CLSM.

#### 2.18.2 IF staining of tissue sections

Sections were subjected to antigen retrieval in ethylenediaminetetraacetic acid (EDTA) buffer (pH 9.0, 100 °C, 5 min), blocked with 3 % BSA, and incubated overnight at 4 °C with CD68 (1:1000, MA5-13324, Invitrogen) and CD206 (1:200, 18704-1-AP, Proteintech) antibodies. After incubation with Alexa Fluor 647– or 568–conjugated secondary antibodies (1:500, Invitrogen) at 37 °C for 1 h, nuclei were counterstained with DAPI (10 min) and visualized by CLSM.

### 2.19 Western blot analysis

Protein expression in MSC-sEVs, cell lysates and rat skin homogenates were quantified using western blotting. Equal amounts of protein were separated on 10 % SDS–PAGE gels and transferred onto PVDF membranes. After blocking with 5 % BSA for 1 h at room temperature, membranes were incubated overnight at 4 °C with primary antibodies: CD9 (20597-1-AP, 1:3000, Proteintech, China), CD81 (27855-1-AP, 1:2000, Proteintech, China), Alix (12422-1-AP, 1:10000 Proteintech, China), calnexin (10427-2-AP, Proteintech, China), iNOS (22226-1-AP, 1:1000, Proteintech, China), CD206 (1:1000, 18704-1-AP, Proteintech, China), IL-6 (A0286,1:1000, ABclonal, China), IL-10 (A12255, 1:1000, ABclonal, China), p38 (A5049,1:1000, ABclonal, China), Phospho-p38 MAPK (PAB43506-P, 1:1000, Bioswamp, China), Phospho-MAPKAPK2 (AP0588, 1:1000, ABclonal, China) and GAPDH (10494-1-AP, 1:5000, Proteintech, China). After three Tris-Buffered Saline with Tween 20 (TBST) washes, membranes were incubated with HRP-conjugated secondary antibodies (SA00001-2, 1:5000, Proteintech, China) and visualized using ECL substrate on a ChemiDoc imaging system (Bio-Rad, USA). Band intensities were quantified with ImageJ and normalized to GAPDH.

### 2.20 Statistical analysis

All data were presented as mean ± standard deviation (SD). Normality was assessed using the Shapiro–Wilk test. Each experiment was independently repeated at least three times. For comparisons among multiple groups, the homogeneity of variance was first tested, followed by one-way analysis of variance (ANOVA) with Tukey's post hoc test. For comparisons between two groups, a two-tailed unpaired Student's *t*-test was used. A *p*-value <0.05 was considered statistically significant. Statistical analyses were performed using GraphPad Prism 9.0 (GraphPad Software, USA).

## 3 Results

### 3.1 Characterization of MSC-sEVs

**Fig. 1:**
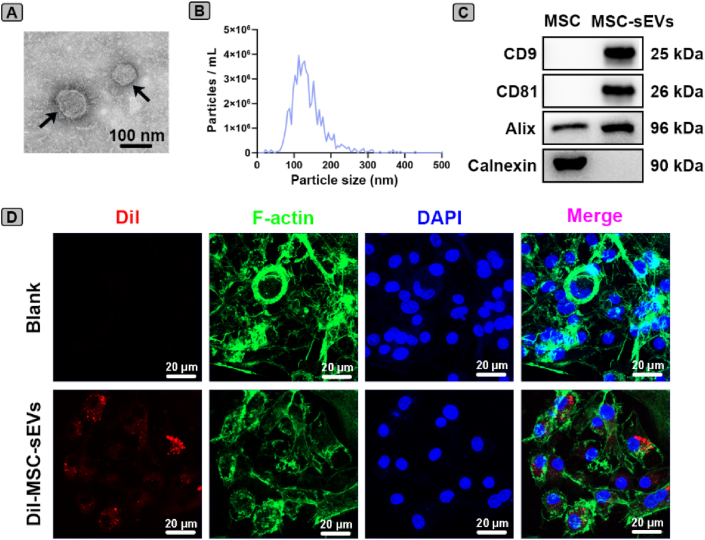
**Characterization of MSC-sEVs**. (A) Representative TEM image of MSC-sEVs. The black arrow indicated the typical vesicle with a bilayer membrane structure. (B) Size distribution profile of MSC-sEVs as determined by NTA. (C) Western blot analysis for MSC-sEVs markers including CD9, CD81, Alix, and Calnexin. (D) Representative confocal images showing the internalization of Dil-labeled MSC-sEVs by BMDMs. MSC-sEVs were labeled with Dil (red), the cytoskeleton was stained with phalloidin (green), and nuclei were counterstained with DAPI (blue). Scale bar, 20 μm. (For interpretation of the references to colour in this figure legend, the reader is referred to the Web version of this article.)

To characterize the MSC-sEVs, we performed TEM, NTA, and western blotting to assess their morphology, size distribution, and protein marker expression, respectively [29,30]. TEM analysis showed that the morphology of MSC-sEVs presented an oval bilayer lipid membrane vesicle (Fig. 1A). NTA analysis demonstrated that MSC-sEVs fell within the expected size range [29], with a peak particle size of approximately 120 nm (Fig. 1B). Western blotting confirmed the presence of exosomal markers CD9, CD81, and Alix, while the absence of Calnexin indicated negligible cellular contamination (Fig. 1C). To further investigate MSC-sEVs uptake, we performed confocal microscopy with Z-stack imaging. As shown in Fig. 1D, red fluorescence of Dil-labeled sEVs colocalized with the green-stained cytoskeleton, confirming that MSC-sEVs were effectively internalized by BMDMs. These results confirmed that MSC-sEVs were successfully isolated and effectively internalized by BMDMs.

### 3.2 MSC-sEVs promoted M1 to M2 macrophage polarization

Diabetic skin along with elevated levels of proinflammatory cytokines and an imbalanced M1/M2 macrophages ratio, which impairs wound healing [31]. The shift of macrophages to M1 or M2 critically regulates inflammation and tissue regeneration [32]. To investigate the effects of MSC-sEVs on macrophage polarization, BMDMs derived from SD rats were first characterized by flow cytometry, over 90 % cells were CD11b^+^CD68^+^, confirming the high purity of the isolated BMDMs (Fig. S2).

**Fig. 2:**
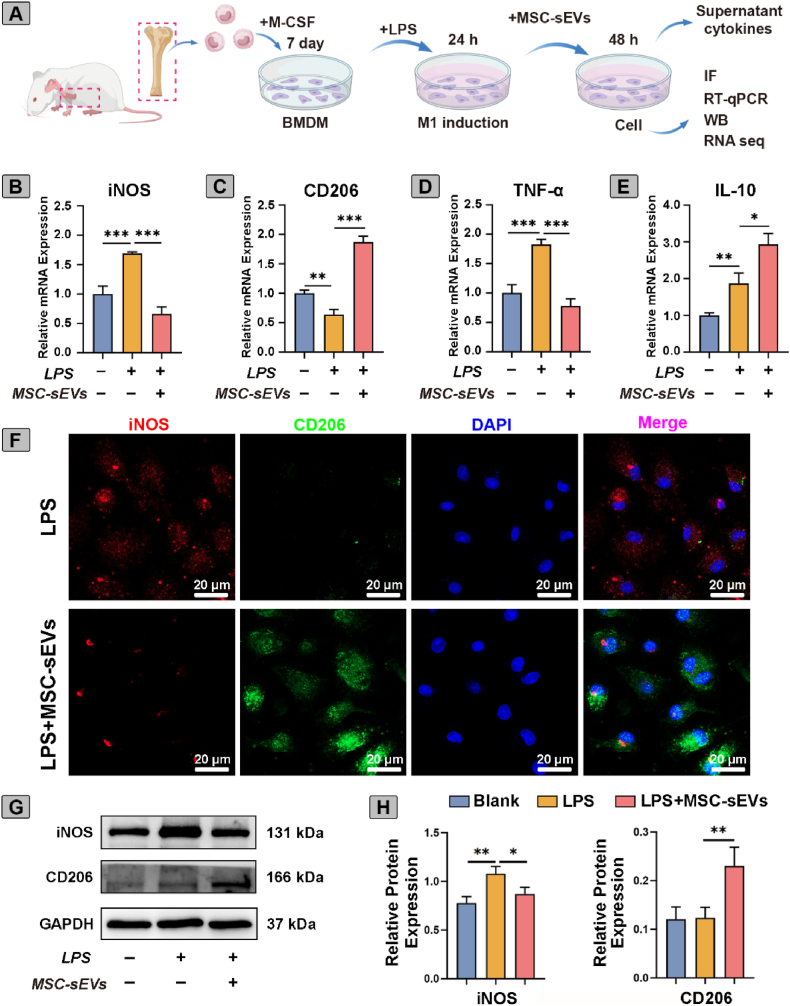
**MSC-sEVs promoted M1 to M2 macrophage polarization.** (A) Schematic diagram of experimental design. (B–E) mRNA expression of iNOS, CD206, TNF-α, and IL-10 in BMDMs treated with or without LPS and MSC-sEVs (n = 3). (F) Representative confocal images stained with iNOS (red), CD206 (green) and DAPI (blue). Scale bar, 20 μm. (G) Western blot analysis and (H) Relative protein expression levels of iNOS and CD206, normalized to GAPDH (n = 3). Data are presented as mean ± SD. ∗*p* < 0.05, ∗∗*p* < 0.01, ∗∗∗*p* < 0.001. (For interpretation of the references to colour in this figure legend, the reader is referred to the Web version of this article.)

As illustrated in Fig. 2A, BMDMs were stimulated with LPS and MSC-sEVs. LPS stimulation markedly increased iNOS and TNF-α, and downregulated CD206 and IL-10. Notably, MSC-sEVs treatment reversed these effects, markedly reducing iNOS and TNF-α expression while enhancing CD206 and IL-10 levels (Fig. 2B–E). Immunofluorescence staining further confirmed these results, showing a predominance of CD206^+^ cells after MSC-sEVs treatment (Fig. 2F). Consistently, western blot analysis demonstrated a significant decrease in iNOS and accompanied by an increase in CD206 following MSC-sEVs treatment (Figure S4, Fig. 2G–H). Collectively, these data indicated that MSC-sEVs effectively facilitate M1-to-M2 macrophage polarization, thereby attenuating inflammatory responses.

### 3.3 MSC-sEVs promoted M2 macrophage polarization via inhibition of the p38 MAPK pathway

**Fig. 3:**
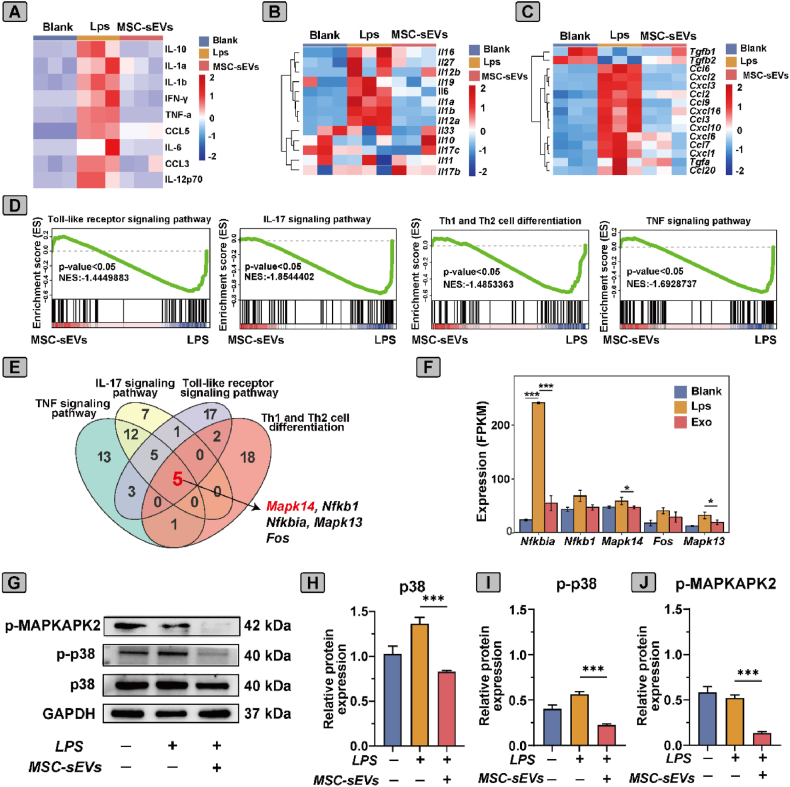
**MSC-sEVs promoted M2 polarization via inhibition of the p38 MAPK pathway.** (A) Heatmap depicting cytokine levels in culture supernatants. (B) Heatmap illustrating the relative inflammatory cytokines levels and (C) Immune response-related genes in different treatment groups. (D) GSEA plots indicating enrichment of key pathway. (E) Venn diagram illustrating the overlap of genes across the pathways, with *Mapk14*, *Nfkb1*, and *Fos* identified as common regulators of inflammation and immune responses. (F) Bar graph showing the expression levels of key genes (n = 3). (G) Western blot analysis of p-MAPKAPK2, p-p38, and total p38 protein expression. (H–J) Quantification of (H) p38, (I) p-p38, and (J) p-MAPKAPK2 levels, demonstrating that MSC-sEVs treatment significantly reduces p-p38 and p-MAPKAPK2 levels. Data are presented as mean ± SD (n = 3). ∗*p* < 0.05, ∗∗∗*p* < 0.001.

To explore how M2 macrophage polarization induced by MSC-sEVs, cytokine assays and RNA-seq were performed. Analysis of culture supernatant revealed that LPS significantly upregulated the pro-inflammatory cytokines, including IL-1β, TNF-α, IFN-γ, IL-6, and CCL3 (Fig. 3A). Treatment with MSC-sEVs markedly attenuated these elevations, indicating a potent anti-inflammatory effect. Transcriptomic profiling revealed pronounced alterations in gene expression across the different treatment groups. Heatmap analysis demonstrated that LPS induced the upregulation of inflammatory cytokine genes, whereas subsequent treatment with MSC-sEVs effectively attenuated this response (Fig. 3B). In addition, genes associated with immune responses were substantially enriched in LPS-treated BMDMs, and their expression was significantly modulated by MSC-sEVs treatment (Fig. 3C).

Gene set enrichment analysis (GSEA) indicated that LPS-stimulated BMDMs markedly upregulated several pro-inflammatory pathways, including Toll-like receptor, IL-17, Th1/Th2 differentiation, and TNF signaling (Fig. 3D). These enrichments were largely reversed following MSC-sEVs treatment, suggesting suppression of inflammatory signaling cascades. A Venn diagram generated from RNA-seq data highlighted overlapping enriched genes across these pathways, with *Mapk14*, *Nfkb1*, and *Fos* identified as key regulators mediating inflammation and immune responses (Fig. 3E). Quantitative analysis further confirmed that MSC-sEVs treatment significantly reduced the expression of *Mapk14* and *Nfkbia* compared to LPS alone (Fig. 3F). To further investigate the mechanisms, western blot analysis focused on the p38 MAPK pathway (Fig. 3G–J). LPS treatment significantly increased the phosphorylation of p38 (p-p38), whereas MSC-sEVs treatment markedly decreased p-p38 and phosphorylated MAPKAPK2 (p-MAPKAPK2) levels. Collectively, MSC-sEVs promoted M2 macrophage polarization through inhibiting p38 MAPK signaling, thereby attenuating LPS-induced inflammation.

### 3.4 Fabrication and characterization of GelMA microspheres

**Fig. 4:**
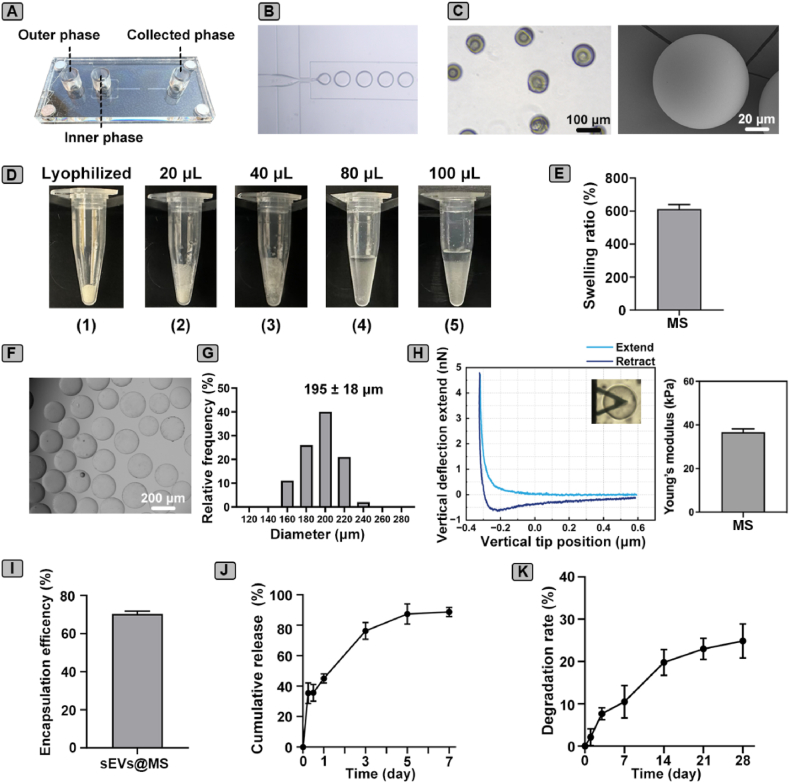
**Fabrication and characterization of GelMA microspheres for MSC-sEVs delivery.** (A) Schematic illustration of the microfluidic chip design for fabricating GelMA hydrogel microspheres. (B) Optical image of water-in-oil droplets formed during the microfluidic process. (C) Morphology of lyophilized GelMA microspheres, with the SEM image shown on the right. (D) Representative images showing the swelling behavior of 10 mg lyophilized microspheres rehydrated with increasing volumes of PBS (20, 40, 80, and 100 μL). (E) Quantitative results of swelling ratio (n = 3). (F) Morphology of rehydrated GelMA microspheres in 100 μL PBS observed under light microscopy, demonstrating restoration to a hydrogel state. (G) Particle size distribution of GelMA microspheres after rehydration. (H) Force-distance curves of individual microspheres measured by AFM and quantitative of Young's modulus (n = 5). (I) Encapsulation efficiency of MSC-sEVs in microspheres (n = 3). (J) Cumulative release profile of MSC-sEVs from GelMA microspheres over 7 days (n = 3). (K) *In vitro* degradation rate of GelMA microspheres over 28 days (n = 4). Data is presented as mean ± SD.

GelMA microspheres were fabricated using a microfluidic chip for the controlled delivery of MSC-sEVs. The microfluidic chip design enabled the generation of uniform water-in-oil droplets, which were subsequently crosslinked to form GelMA microspheres (Fig. 4A and B). The lyophilized microspheres retained their spherical morphology and structural integrity, as confirmed by imaging (Fig. 4C). Swelling behavior was assessed by hydrating the microspheres with increasing volumes of PBS until a well-dispersed hydrogel suspension was obtained (Fig. 4D). The swelling ratio reached 613.3 ± 26.3 %, indicating a high water absorption capacity (Fig. 4E). Upon rehydration, the lyophilized GelMA microspheres restored their hydrogel state, forming spherical microspheres as observed under light microscopy (Fig. 4F). Particle size analysis revealed a mean diameter of 195 ± 18 μm, with the majority of particles ranging from 160 to 240 μm (Fig. 4G). AFM measurements showed that the Young's modulus of rehydrated microspheres was about 37 ± 1.6 kPa (Fig. 4H). In line with other research, 7.5 % GelMA microspheres exhibited an elastic modulus of approximately 15 ± 2 kPa when measured by AFM nanoindentation and validated by bulk compression testing was 14 ± 2 kPa [33]. The higher stiffness observed in our study is likely attributable to the increased GelMA concentration (10 %), consistent with the known positive correlation between GelMA polymer content and mechanical strength. For MSC-sEVs encapsulation, 10 mg lyophilized microspheres were rehydrated with 100 μL MSC-sEVs suspension, and the encapsulation efficiency was 70 ± 2 % (Fig. 4I). The release kinetics of MSC-sEVs from the microspheres exhibited a sustained pattern, with 45 % ± 3 % released on day 1, 76 ± 6 % on day 3, and reaching 89 % ± 3 % by day 7. (Fig. 4J). The release of MSC-sEVs from sEVs@MS can be internalized by BMDMs and promoted M2 polarization (Fig. S3). The degradation behavior of rehydrated microspheres was monitored over 28 days, showing gradual mass loss over time (Fig. 4K).

Collectively, these findings demonstrated that GelMA microspheres, with their uniform size, appropriate degradation rate and mechanical properties, make them a promising platform for the controlled delivery of MSC-sEVs in wound repair applications.

### 3.5 MSC-sEVs treatment enhanced wound healing in diabetic rats

**Fig. 5:**
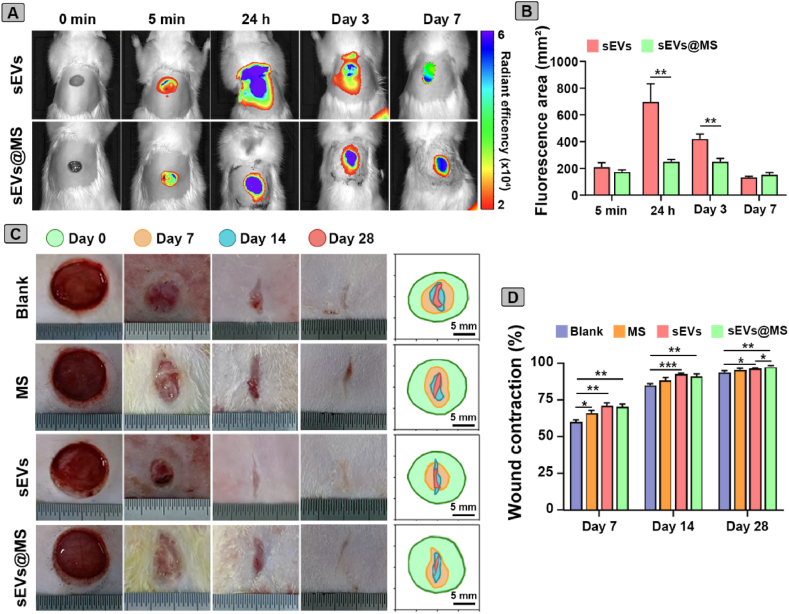
***In vivo* assessment of biodistribution and wound healing effects of MSC-sEVs treatment.** (A) Representative images showing the biodistribution of DiR-labeled sEVs and sEVs@MS at different time points. (B) Quantification of fluorescence area, illustrating the release of sEVs and sEVs@MS (n = 3). (C) Representative images of wound closure. Wound closure traces are indicated for day 0 (green), 7 (orange), 14 (blue), and 28 (red). (D) Quantitative analysis of wound contraction rates for each treatment group. Data are presented as mean ± SD (n = 3). ∗*p* < 0.05, ∗∗*p* < 0.01, ∗∗∗*p* < 0.001. (For interpretation of the references to colour in this figure legend, the reader is referred to the Web version of this article.)

*In vivo* biodistribution of sEVs was evaluated using real-time fluorescence imaging analysis (Fig. 5A). Fluorescence signals from DiR-labeled sEVs and sEVs@MS revealed their localization and retention. In the sEVs group, the fluorescence signal at the wound bed gradually decreased over time, whereas the sEVs@MS group maintained a persistent fluorescent signal, indicating prolonged retention of sEVs at the wound bed. Quantitative analysis revealed that the fluorescence area of sEVs (697 ± 136 mm^2^ at 24 h, 420 ± 36 mm^2^ on day 3) was significantly larger than that of sEVs@MS (251 ± 18 mm^2^ at 24 h, 251 ± 24 mm^2^ on day 3) (Fig. 5B). These results indicated that sEVs@MS enhanced local retention and sustained release of sEVs from microspheres.

The therapeutic effects of sEVs and sEVs@MS were evaluated in a full-thickness skin wound model of TIDM rats. Digital imaging and wound contraction measurements over 28 days post-injury revealed significantly enhanced healing in both sEVs-treated groups (Fig. 5C and D). By day 3, all groups showed initial wound closure (Fig. S5). On day 7, wound contraction in the sEVs and sEVs@MS groups reached nearly 70 %, significantly higher than in the blank group. By day 14, the difference was more pronounced: the sEVs group achieved almost complete wound closure, whereas the blank group retained ∼15 % of the original wound area. By day 28, sEVs@MS group were almost completely healed, with regenerated smooth skin. Collectively, these results indicated that MSC-sEVs treatment promoted diabetic wound healing in both the sEVs and sEVs@MS groups. Notably, the encapsulation of MSC-sEVs within microspheres enabled localized and sustained release.

### 3.6 MSC-sEVs treatment enhanced M2 polarization and reduced inflammation in diabetic wounds

**Fig. 6:**
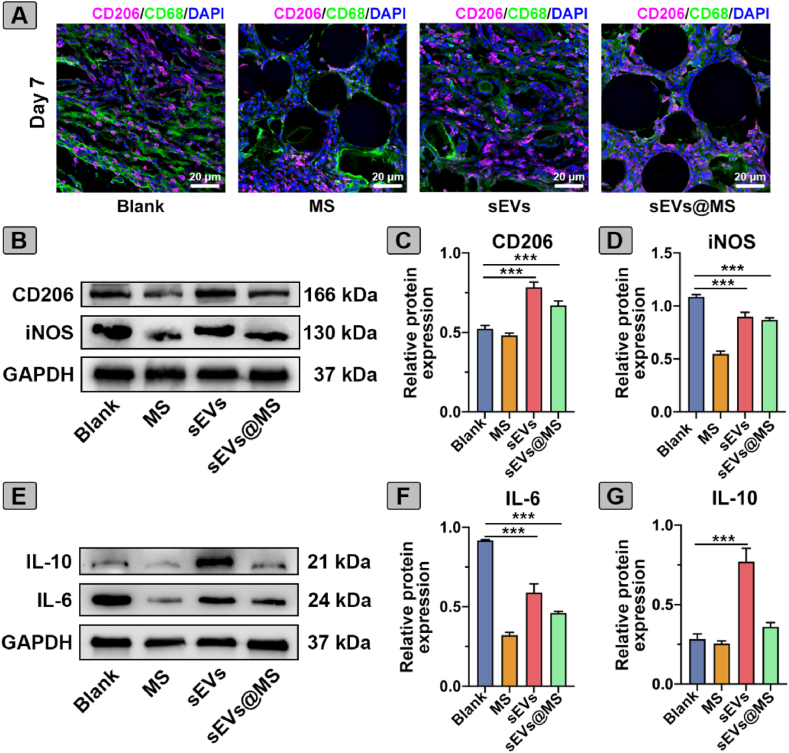
**MSC-sEVs treatment enhanced M2 macrophage polarization and suppresses inflammation in diabetic wounds.** (A) IF staining of CD206 and CD68 on day 7. Scale bar, 20 μm. (B) Western blot analysis of macrophage polarization markers CD206 and iNOS in regenerated wound tissues. (C–D) Quantification of relative protein expression levels of (C) CD206 and (D) iNOS, normalized to GAPDH (n = 3). (E) Western blot analysis of cytokine expression (IL-10 and IL-6) in the regenerated tissue. (F–G) Quantification of relative protein expression levels of (F) IL-6 and (G) IL-10, normalized to GAPDH. Data are presented as mean ± SD (n = 3). ∗∗∗*p* < 0.001.

To assess MSC-sEVs treatment on macrophage polarization and inflammation in diabetic wounds, IF staining of CD206 and CD68 was performed on day 7 (Fig. 6A). The sEVs increased CD206 expression, indicating a partial shift toward M2 polarization. Notably, in the sEVs@MS group, a greater number of CD206-positive macrophages were observed surrounding the microspheres, suggesting that sustained release of sEVs from the microspheres effectively promotes M2 macrophage polarization.

Western blot analysis corroborated these observations, showing markedly increased CD206 levels and significantly reduced iNOS expression in the sEVs and sEVs@MS groups relative to the blank group (Fig. 6B–D). Cytokine profiling further supported the anti-inflammatory effects, with IL-6 levels decreased and IL-10 levels elevated in both sEVs-treated groups (Fig. 6E–G). Collectively, these results demonstrated that MSC-sEVs treatment enhanced M2 macrophage polarization and suppressed inflammation in diabetic wounds.

### 3.7 sEVs@MS enhanced tissue regeneration in diabetic wounds

**Fig. 7:**
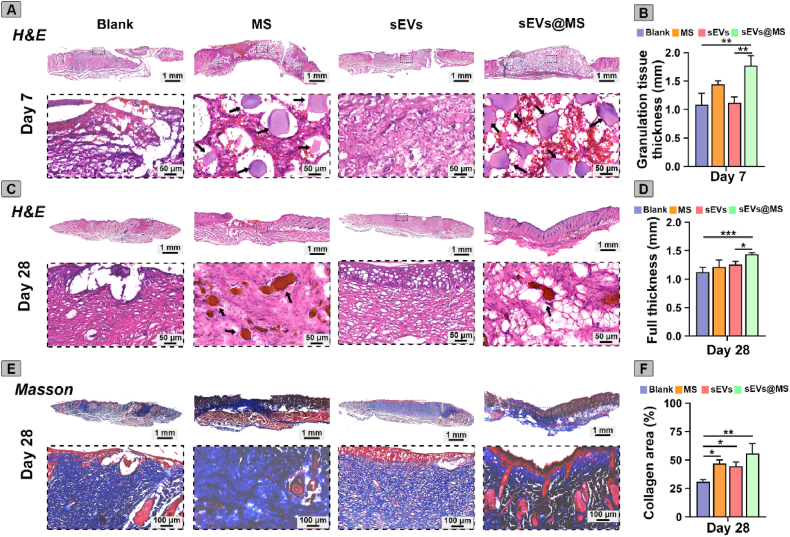
**Histological analysis of tissue regeneration.** (A) H&E staining showing wound regeneration in the Blank, MS, sEVs, and sEVs@MS groups on day 7, and (C) on day 28 post-wounding. Black arrows indicate microspheres present in the wound bed. Scale bars: 1 mm (upper) and 50 μm (lower). (B) Quantification of granulation tissue thickness at the center of wounds on day 7. (D) Quantification of the full thickness of regenerated skin tissue after different treatments on day 28. (n = 3 per group). (E) Masson's trichrome staining of skin sections on day 28 post-wounding. Scale bars: 1 mm (upper) and 100 μm (lower). (F) Quantitative analysis of collagen area on day 28. Data are presented as mean ± SD (n = 3). ∗*p* < 0.05, ∗∗*p* < 0.01, ∗∗∗*p* < 0.001.

On day 7, H&E staining showed disorganized tissue structure and limited re-epithelialization in the blank group (Fig. 7A). MS and sEVs@MS groups displayed enhanced cellular infiltration and more structured granulation tissue (Fig. 7B), likely attributed to the microspheres providing structural support for nascent tissue formation. The sEVs@MS group, in particular, showed microspheres tightly embedded within the granulation tissue and well-formed tissue architecture.

By day 28, histological analysis further highlighted the differential effects of treatment. H&E staining showed incomplete re-epithelialization in the blank group, whereas the sEVs group exhibited advanced re-epithelialization with organized tissue architecture. Notably, the sEVs@MS group achieved complete re-epithelialization and a well-organized dermis, indicating enhanced skin regeneration (Fig. 7C and D). Masson's trichrome staining further confirmed these findings (Fig. 7E and F). The blank group displayed sparse and disorganized collagen deposition, the sEVs group showed denser and more aligned collagen fibers, and the sEVs@MS group demonstrated the most robust collagen deposition with well-aligned, mature fibers and a highly organized extracellular matrix.

Collectively, these results indicated that MSC-sEVs treatment, particularly sEVs@MS, promoted tissue regeneration in diabetic wounds.

## 4 Discussion

DFUs are one of the major reasons for amputation among patients with diabetes mellitus [34]. Current hydrogel dressings are hard to address the complex therapeutic needs of these chronic wounds adequately [35]. In this study, we engineered GelMA microspheres as a hydrogel dressing for MSC-sEVs delivery, enabling sustained release of these therapeutic vesicles. Our results demonstrated that MSC-sEVs effectively inhibit the p38 MAPK signaling pathway, facilitating macrophage polarization from M1 toward M2. This immunomodulatory effect significantly ameliorated the inflammatory microenvironment at the wound site, thereby enhancing wound healing in TIDM rat models.

Hyperglycemia associated with diabetes impairs macrophages M1-to-M2 transformation, resulting in chronic inflammation and delayed wound recovery [11,12]. MSC-sEVs are able to modulate macrophage polarization and support wound healing through the delivery of bioactive cargo such as microRNAs and proteins [6,36]. Recent studies have demonstrated that MSCs can facilitate this phenotypic switch during the early inflammatory phase. For example, MSC-sEVs enriched with miR-27-3p can promote M2 polarization by targeting CSF-1 [37]. Engineered exosomes delivering miR-146a enhance wound repair in diabetic mice by silencing IRAK1 [38], and miR-182-loaded sEVs modulated macrophage phenotype via the suppression of TLR4 and subsequent inhibition of the NF-κB pathway [39]. These findings support our study, suggesting that bioactive components within MSC-sEVs can promote BMDMs M1-to-M2 conversion, and modulate cytokines secretion.

Macrophage polarization is complex process regulated by multiple signaling pathways, including PI3K/AKT, JAK/STAT, and NF-κB [9]. Recent evidence also highlights the involvement of NF-κB and MAPK pathway in macrophage M2 polarization [40]. In this study, we found that MSC-sEVs effectively promoted macrophage M2 polarization by inhibiting p38 MAPK pathway. LPS activates TLR4, leading to downstream NF-κB and MAPK signaling and upregulation of pro-inflammatory genes [41,42]. We found that MSC-sEVs suppressed LPS-induced upregulation of p38 and MAPKAPK2 phosphorylation level in macrophage, highlighting the role of p38 MAPK pathway in modulating macrophage polarization. The immunoregulatory function of MSC-sEVs is largely attributed to their cargo, particularly microRNAs. For example, MSC-sEVs derived let-7b inhibited TLR4 expression, thus reducing the downstream activation of MKK3/6 and decreasing p38 phosphorylation [43], while miR-21 delivered by MSC-sEVs downregulates p38 MAPK to modulate β-cell apoptosis in diabetes [44]. These studies support our observations that MSC-sEVs reduce p-p38 and p-MAPKAPK2 levels, providing a mechanistic basis for their effect on macrophage polarization.

Hydrogels are emerging as smart delivery systems to enhance the immunomodulatory potential of MSC-sEVs. It was demonstrated that M2 macrophage derived-exosomes released from hydrogels confer protective effects in diabetic wound healing [21], while GelMA hydrogels delivering sEVs enriched with miR-17-5p promote healing by downregulating phosphatase and tensin homolog in dermal fibroblasts [20]. Similarly, GelMA-encapsulated extracellular vesicles loaded with VH298 enhance angiogenesis in endothelial cells, facilitating diabetic wound repair [45]. Developing biomaterials with immunomodulatory therapeutic effects is a promising strategy in DFUs treatment. These findings support our design of MSC-sEVs-loaded microspheres, which provide both structural support and sustained release to enhance anti-inflammatory and regenerative effects in diabetic wounds.

## 5 Conclusion

This study showed that MSC-sEVs can suppress inflammation through promoting M1 to M2 macrophage polarization by inhibiting the p38 MAPK signaling pathway. MSC-sEVs encapsulated in GelMA microspheres enable sustained release and prolonged retention of MSC-sEVs at the wound site. By combining the immunomodulatory effects of MSC-sEVs with GelMA microspheres dressing, the sEVs@MS markedly accelerated wound healing, promoted collagen formation, and supported epidermal regeneration in diabetic rats. This study provides a promising therapeutic strategy for diabetic wound healing. To build upon our findings, further molecular mechanism exploration thoroughly investigating the active components within MSC-sEVs is required in future studies.

## CRediT authorship contribution statement

**Weizhao Li:** Writing – original draft, Data curation. **Jiajia Chen:** Software, Investigation, Data curation. **Lu Yu:** Validation, Software, Methodology. **Lu Ding:** Visualization, Validation. **Xiaoying Zhang:** Supervision. **Leping Yan:** Supervision, Resources, Funding acquisition. **Ming Shi:** Writing – review & editing, Resources, Funding acquisition, Conceptualization.

## Declaration of competing interest

The authors declare that they have no known competing financial interests or personal relationships that could have appeared to influence the work reported in this paper.

## Data Availability

The RNA-seq data generated in this study have been deposited in Mendeley Data [https://doi.org/10.17632/gm29wd7b94.1] and are publicly available. Other data supporting the findings of this study are available from the corresponding author upon reasonable request.
