# Supplementary material for: Mesenchymal stem cell-derived small extracellular vesicles-loaded GelMA microspheres enhance diabetic wound healing by promoting M2 macrophage polarization through p38 MAPK inhibition
Source: Mater Today Bio. 2025 Oct 17;35:102423. doi: 10.1016/j.mtbio.2025.102423 (PMC12590144; doi:10.1016/j.mtbio.2025.102423)
Supplement: Multimedia component 1 [file mmc1.docx]

**Supplemental information for**

Mesenchymal stem cell-derived small extracellular vesicles-loaded GelMA microspheres enhance diabetic wound healing by promoting M2 macrophage polarization through p38 MAPK inhibition

Weizhao Li ^a, b †^, Jiajia Chen^a, †^, Lu Yu ^c, †^, Lu Ding ^a^, Xiaoying Zhang ^d^, Leping Yan ^a, e *^, Ming Shi ^a, *^


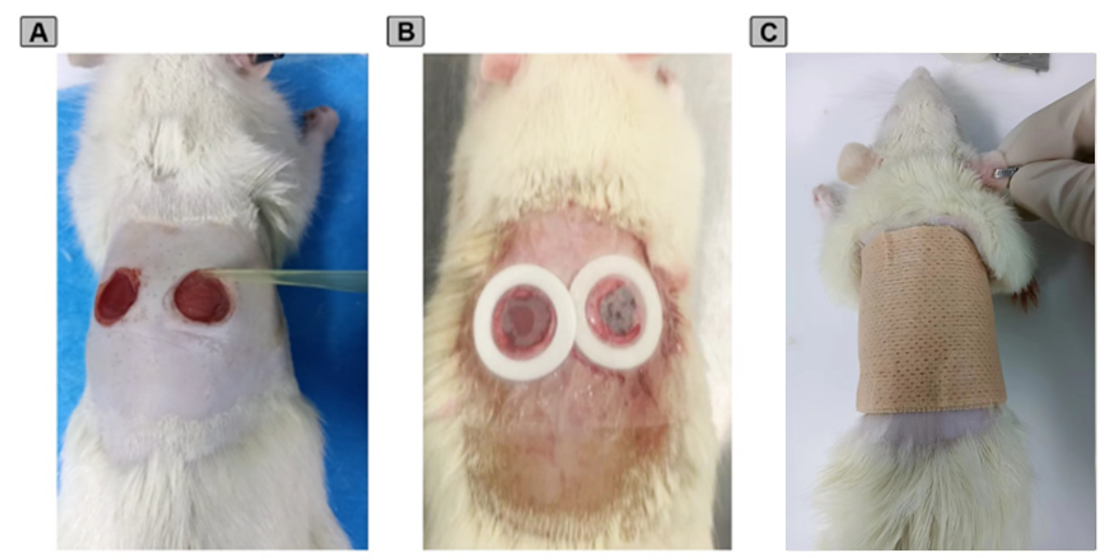


**Figure S1. Fixation method of the rehydrated GelMA microspheres on the wound.** (A) The rehydrated microsphere dressings of MS and sEVs@MS group were evenly applied and filled in the wound area. (B) Wounds were covered with a sticker with a rubber splint (inter diameter ~15 mm) covered by transparent and waterproof film. (C) Wounds areas were then secured with a self-adhesive bandage to prevent sticker detachment.


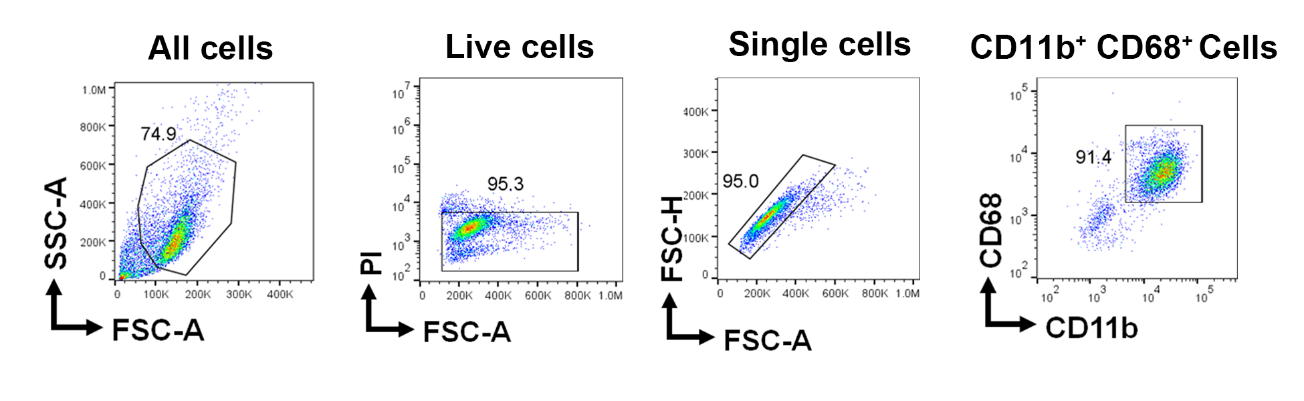


**Figure S2.** **Identification of BMDMs.** Flow cytometry analysis confirmed over 90% of the differentiated cells were double-positive for CD11b and CD68, confirming the successful isolation and differentiation of BMDMs.


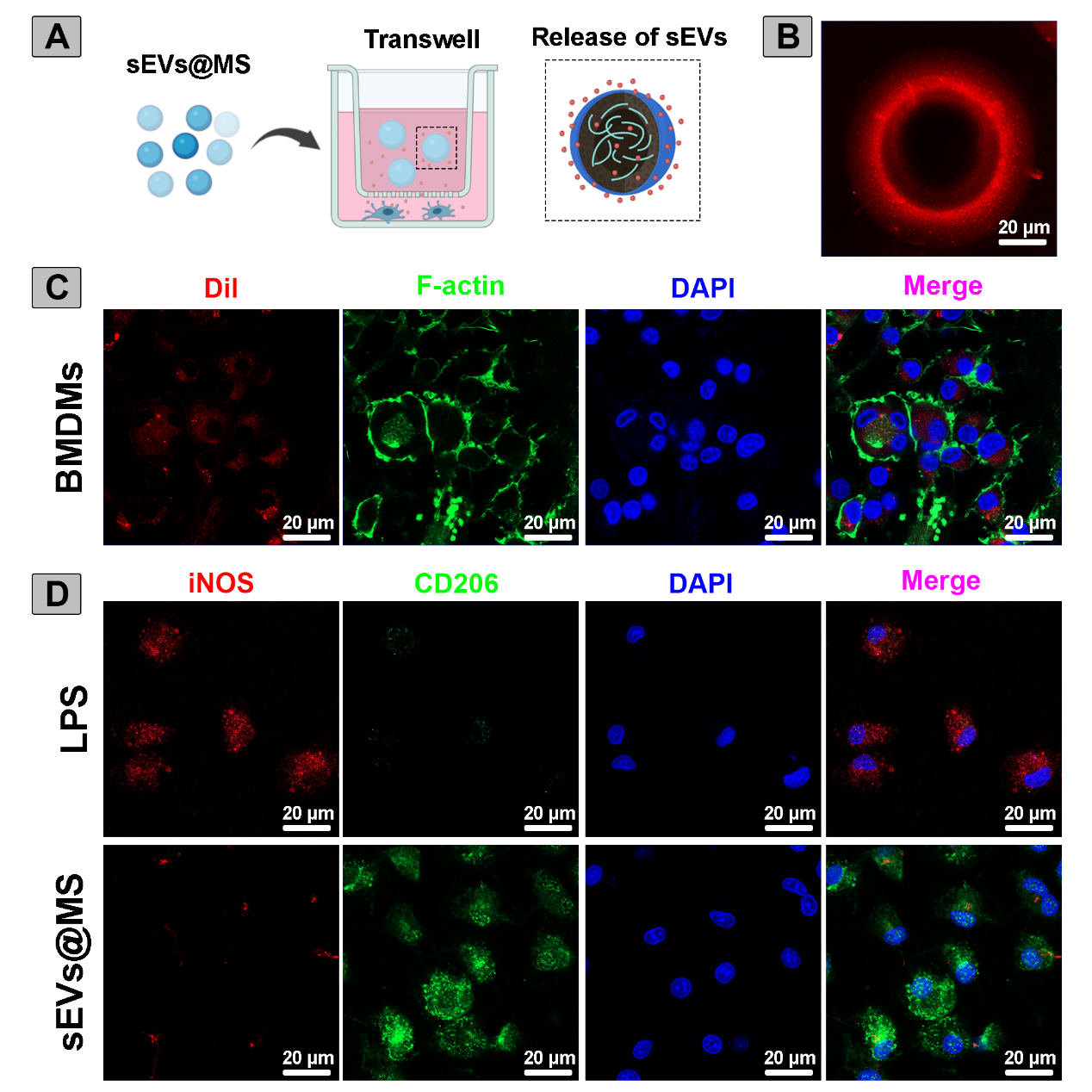


**Figure S3.** **MSC-sEVs released by sEVs@MS promoted M2 polarization *in vitro*.** (A) Schematic illustration of the Transwell co-culture system, in which BMDMs were co-cultured with MSC-sEV-loaded microspheres (sEVs@MS). (B) Fluorescence image showing the distribution of DiI-labeled MSC-sEVs within the microspheres. (C) Representative confocal images demonstrating the internalization of DiI-labeled MSC-sEVs (released from microspheres) by BMDMs. Scale bar: 20 μm. (D) Immunofluorescence staining of iNOS (red), CD206 (green), and nuclei (DAPI, blue), with merged images indicating that sEVs@MS promote M2 macrophage polarization. Scale bar: 20 μm.


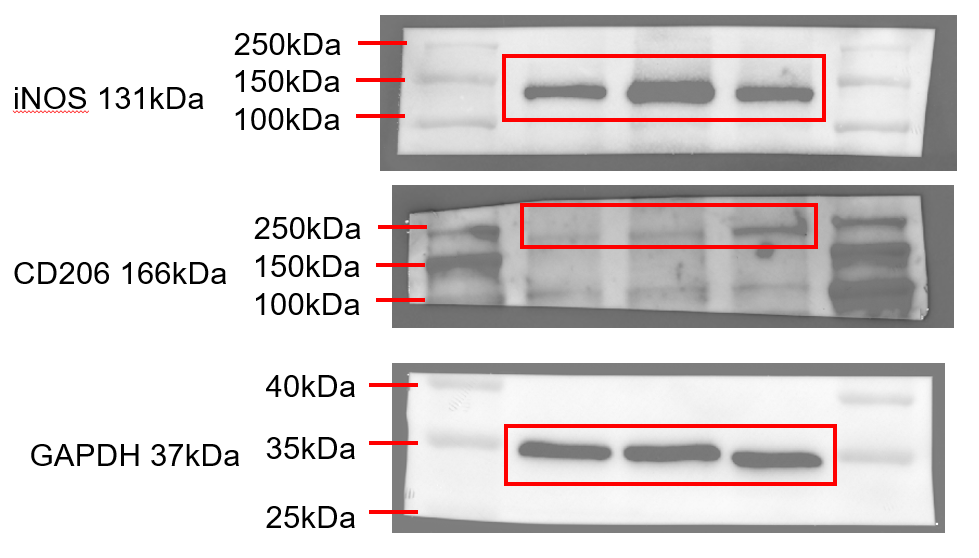


**Figure S4. Original unprocessed Western blot images of Figure 2G.**


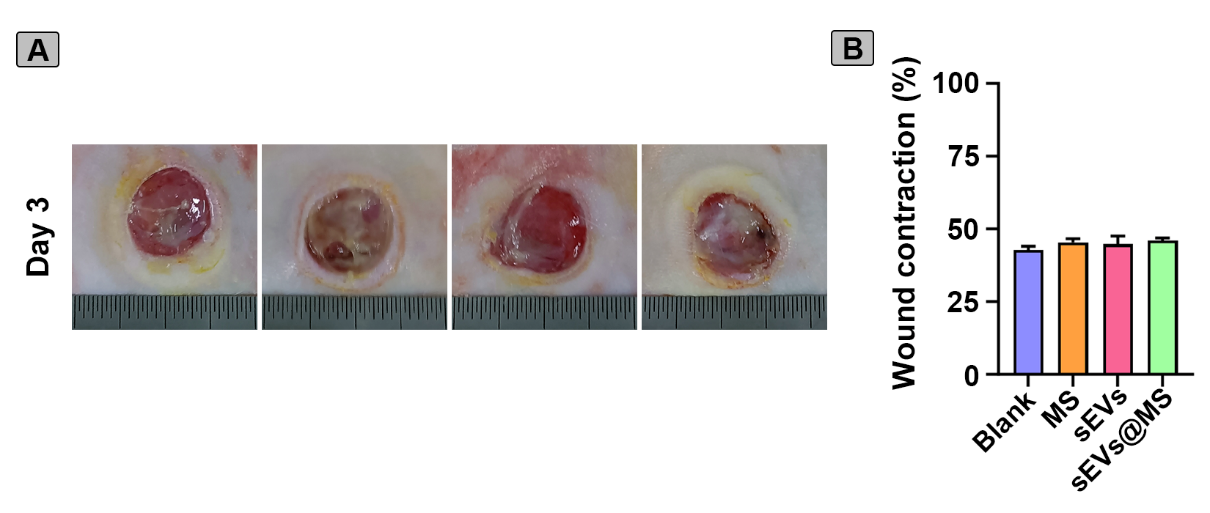


**Figure S5. Wound closure traces on day 3.** (A) Representative images of wound closure in rats treated with blank, MS, sEVs, and sEVs@MS on day 3. (B) Quantitative analysis of wound contraction rates for each treatment group on day 3. Data are presented as mean ± SD (n = 3).
